# Four Cases of Calcium Pyrophosphate Deposition Disease Presenting With Polymyalgia-Like Symptoms and Chondrocalcinosis in the Shoulder and Hip Joints Identified on CT Imaging

**DOI:** 10.7759/cureus.76897

**Published:** 2025-01-04

**Authors:** Ryosuke Ono, Ken Horibata

**Affiliations:** 1 Department of Internal Medicine, Kameyama Municipal Medical Center, Kameyama, JPN; 2 Department of Kameyama Community Medicine, Mie University School of Medicine, Tsu, JPN

**Keywords:** calcium pyrophosphate deposition disease (cppd), chondrocalcinosis, crown dense syndrome, pmr, polymyalgia, polymyalgia rheumatica, pseudo-gout, rheumatoid arthritis, seronegative rheumatoid arthritis

## Abstract

Calcium pyrophosphate deposition (CPPD) disease is a rheumatic disorder frequently observed in elderly individuals, often presenting with symptoms resembling polymyalgia rheumatica (PMR). Despite differing treatment strategies for these conditions, a definitive method for distinguishing between them remains unestablished. We report the cases of four elderly patients initially presenting with polymyalgia (PM), ultimately diagnosed with CPPD disease. Early-stage CT scans in all cases demonstrated chondrocalcinosis (CC) in the shoulder and hip joints. These findings suggest that PM in CPPD disease is attributable to calcium pyrophosphate deposition in these joints. Moreover, the identification of CC via CT imaging may aid in differentiating CPPD disease with PM from true PMR. Further research is warranted to refine this diagnostic distinction.

## Introduction

Polymyalgia rheumatica (PMR) is characterized by inflammatory pain and morning stiffness in the shoulders and pelvic girdle, predominantly affecting individuals aged 50 years and older. It is the most common rheumatic disorder among the elderly, but several other conditions can present with polymyalgia (PM)-like symptoms resembling PMR [1,2].

Calcium pyrophosphate deposition (CPPD) disease is one such condition. It is caused by the deposition of calcium pyrophosphate (CPP) crystals in articular cartilage and surrounding tissues [2]. These crystal deposits, termed chondrocalcinosis (CC), appear as linear or punctate calcifications on plain X-ray or CT imaging [3]. While the most common manifestation of CPPD disease is pseudogout, characterized by acute episodic monoarthritis, other forms of CPPD disease can also occur. McCarty’s 1975 classification described polymyalgia-like phenotypes, such as pseudo-rheumatoid arthritis and pseudo-polymyalgia rheumatica [4]. In 2012, the European League Against Rheumatism (EULAR) task force introduced standardized terminology, categorizing chronic CPP crystal inflammatory arthritis presenting with polymyalgia (CPPD/PM) [5]. Furthermore, crown dense syndrome (CDS), a variant of CPPD, has also been reported to present with polymyalgia [6].

The frequency of polymyalgia in CPPD disease remains uncertain. Studies suggest that 30%-50% of patients initially diagnosed with PMR, after excluding rheumatoid arthritis (RA) and spondylarthritis, also have concurrent CPPD disease [7,8]. However, distinguishing CPPD/PM from PMR is challenging due to the lack of a definitive diagnostic standard for either condition. The prevalence of asymptomatic CC in elderly individuals, estimated at 7% by Neame et al. [9], further complicates this distinction, as early-stage CPPD/PM may be misdiagnosed as PMR. Accurate differentiation is critical, given the differences in treatment and prognosis. Misdiagnosing CPPD/PM as PMR can lead to inappropriate steroid overuse.

We present four cases of patients with PM in whom CT imaging revealed CC in major joints, ultimately leading to a diagnosis of CPPD disease. This report explores the potential utility of detecting CC in major joints as a means to differentiate CPPD/PM from PMR, thereby informing appropriate treatment strategies.

## Case presentation

Case 1

An 86-year-old female patient with a 12-year history of RA managed with 5 mg/day of prednisolone was hospitalized at our institution for multiple compression fractures of the thoracolumbar vertebrae following a fall. During her admission, the orthopedic specialist discontinued prednisolone.

**Figure 1:**
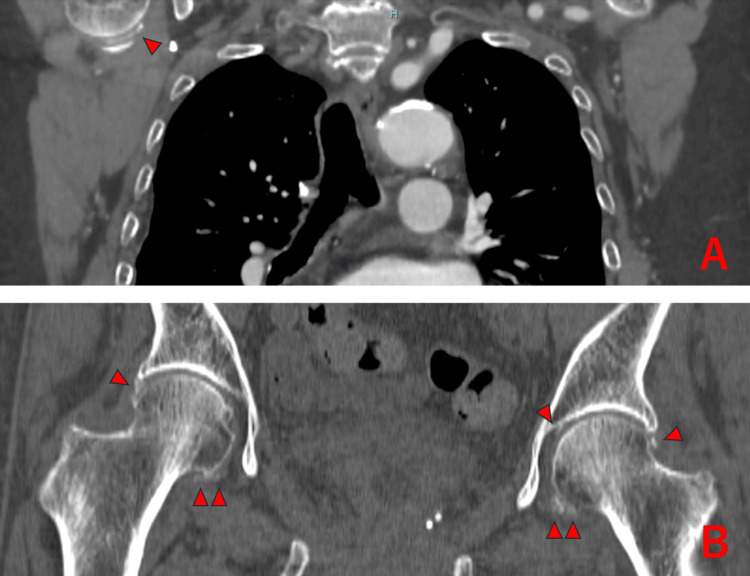
Right shoulder and bilateral hip joints of Case 1 showing chondrocalcinosis (△).

On the 11^th^ day of hospitalization, the patient developed chest symptoms, and contrast-enhanced CT revealed mild pulmonary embolism and distal deep vein thrombosis, prompting anticoagulation therapy. The same CT scan showed CC in both shoulder and hip joints, as well as calcifications in the pubic symphysis, ischial tendon, and greater trochanter tendon (Figure 1).

On the 21^st^ day, she experienced left hip pain, which resolved after seven days of treatment with celecoxib 200 mg/day for pseudogout. On the 50^th^ day, right knee pain developed and was treated with celecoxib for nine days, although joint aspiration was not performed. On the 68^th^ day, bilateral shoulder pain and recurrent right knee pain appeared. Blood tests revealed elevated inflammatory markers and liver dysfunction (white blood cell (WBC) count of 10,500/µL, C-reactive protein (CRP) of 18.6 mg/dL, erythrocyte sedimentation rate (ESR) of 98 mm/hr, aspartate aminotransferase (AST) of 62 U/L, alanine aminotransferase (ALT) of 11 U/L, lactate dehydrogenase (LDH) of 242 U/L, alkaline phosphatase (ALP) of 870 U/L, and gamma-glutamyl transpeptidase (γ-GTP) of 413 U/L). Abdominal CT showed no abnormalities apart from asymptomatic gallstones.

Negative results for rheumatoid factor and anti-CCP antibodies supported a diagnosis of PMR. Prednisolone 15 mg/day was initiated, resolving joint pain and normalizing inflammatory markers within two weeks. Liver enzyme levels returned to normal, and the patient was discharged on the 102^nd^ day of hospitalization. Post discharge, the prednisolone dose was tapered to 5 mg/day over six months without recurrence of arthritis or inflammation.

At 22 months post discharge, the patient presented with severe neck pain and mild bilateral hip pain. Blood tests indicated elevated CRP, suggesting inflammation, and prednisolone was increased to 10 mg/day, leading to symptom resolution. The dose was subsequently tapered to 5 mg/day.

At 37 months post discharge, the patient experienced a recurrence of polymyalgia with lower extremity edema. Prednisolone was increased to 15 mg/day, resolving symptoms. However, symptoms recurred at a reduced dose of 10 mg/day. Methotrexate 6 mg/week was introduced but proved ineffective. Further evaluation revealed primary hyperparathyroidism with hypercalcemia due to a parathyroid tumor. Suspecting CPPD disease, meloxicam 10 mg/day was initiated, leading to symptom resolution. Prednisolone 5 mg/day and meloxicam 10 mg/day were continued.

At 51 months post discharge, the patient developed left knee arthritis. Joint aspiration confirmed the presence of CPP crystals, establishing a diagnosis of CPPD/PM. Colchicine 0.5 mg/day was initiated, and the patient has remained symptom-free for over two years.

Case 2

An 86-year-old male patient with a history of diabetes mellitus and hypertension was under regular care at another clinic. Approximately 40 days prior to presentation, he developed acute pain and stiffness in the neck, shoulders, and hips. After consulting a nearby orthopedic clinic, he was prescribed loxoprofen 60 mg three times daily for one week. Although his symptoms temporarily improved, they recurred after discontinuing the medication. During a routine visit to his primary care clinic, anorexia and anemia of chronic disease were detected, leading to his referral to our hospital.

**Figure 2:**
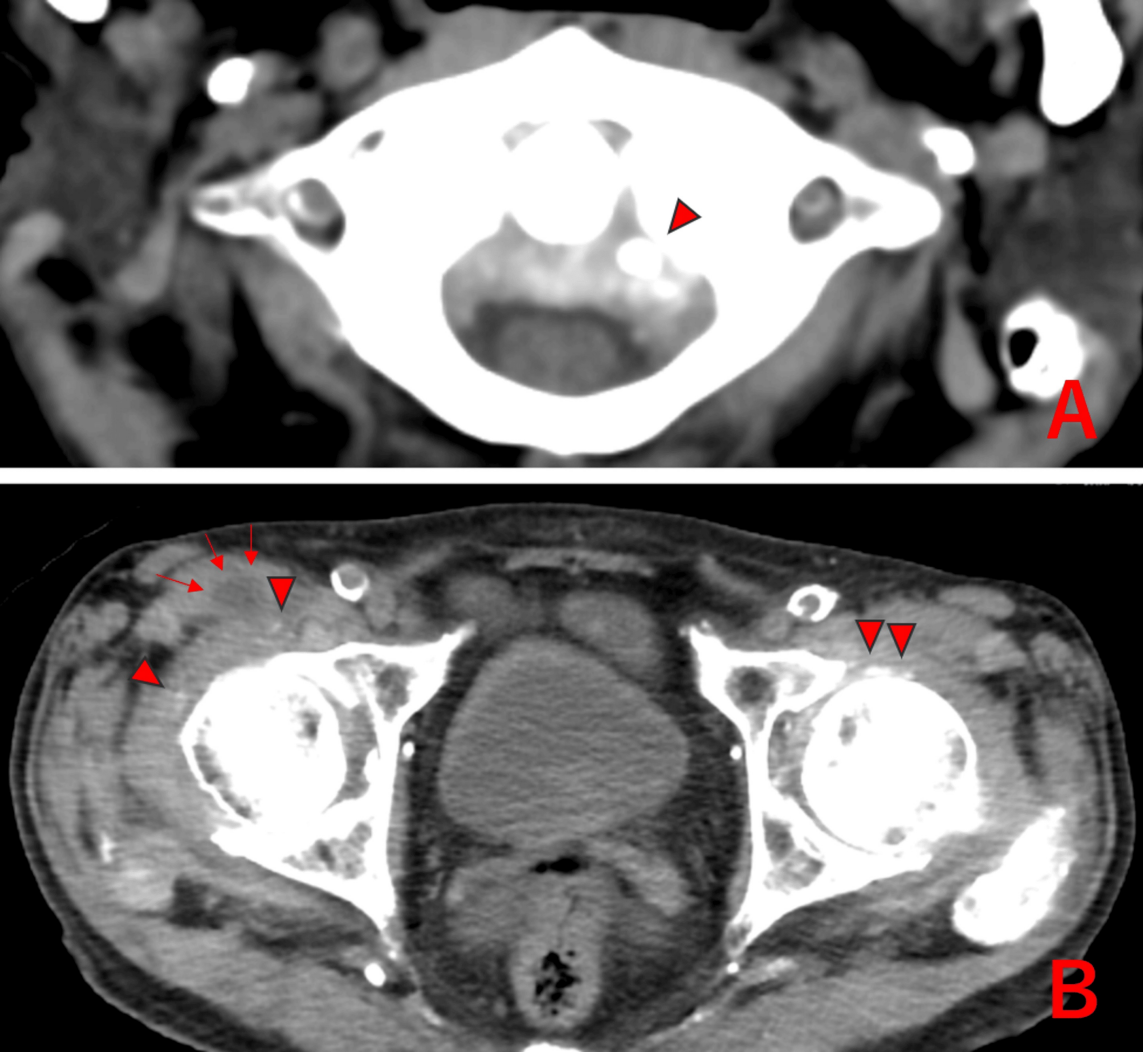
Axial vertebral region and bilateral hip joints of Case 2 showing chondrocalcinosis (△); fluid accumulation observed in the right hip bursa (↑).

On initial evaluation, the patient reported severe neck pain with rotation and motion-related pain in the hips, but no motion-related shoulder pain or peripheral arthritis was observed. Laboratory findings revealed elevated inflammatory markers (WBC of 11,000/µL, CRP of 13.5 mg/dL, and ESR of 112 mm/hr), while other biochemical parameters were within normal limits. Rheumatoid factor was positive at 75 U/mL, but anti-CCP antibodies were negative. Plain CT imaging showed CC around the atlantoaxial joint, joint effusion in the right hip, and CC within both hip joints (Figure 2).

A diagnosis of CPPD/PM was made, and treatment with prednisolone 15 mg/day was initiated. Within two weeks, the patient experienced significant symptom relief, and inflammatory markers normalized. Prednisolone was subsequently discontinued, and maintenance therapy with colchicine 0.5 mg/day and meloxicam 10 mg/day was prescribed. The patient has remained symptom-free during six months of follow-up.

Case 3

An 80-year-old female patient with a history of mild cognitive impairment underwent mechanical valve replacement and pacemaker implantation for aortic valve stenosis at the age of 60.

One month prior to admission, she developed fatigue, decreased activities of daily living, lower back discomfort, and lower extremity edema. Her primary care physician was unable to identify the cause, prompting her referral to our hospital.

**Figure 3:**
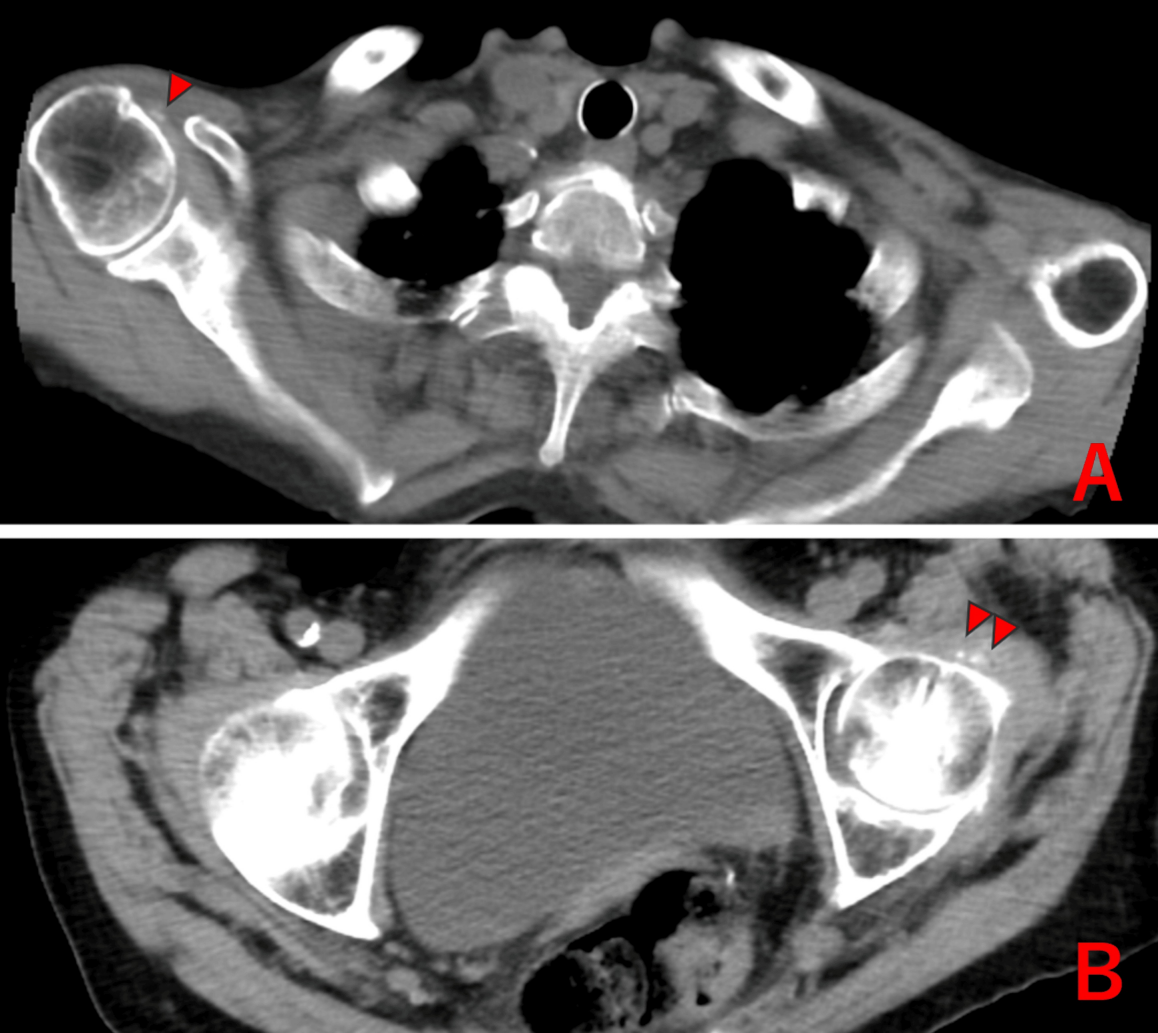
Right shoulder and left hip joint of Case 3 showing chondrocalcinosis (△)

At the initial visit, she reported motion-related pain in both shoulders and moderate lower extremity edema. However, there was no neck pain during rotation, motion-related pain in the hips, or peripheral arthritis. Laboratory tests showed elevated inflammatory markers (WBC of 8,100/µL, CRP of 3.8 mg/dL, and ESR of 67 mm/hr), with negative rheumatoid factor and anti-CCP antibodies. Plain CT revealed CC in the right shoulder and left hip joints (Figure 3). Additionally, high BNP levels, pulmonary edema on imaging, and prosthetic valve restenosis findings suggested heart failure, necessitating hospitalization.

Diuretic therapy improved her lower extremity and pulmonary edema, but her symptoms and elevated inflammatory markers persisted. Ultrasound revealed bilateral shoulder bursitis without evidence of infection. She was diagnosed with PMR and treated with prednisolone 15 mg/day, resulting in symptom resolution and normalization of inflammatory markers. She was discharged on hospital day 24.

Following discharge, her CRP levels fluctuated between 1.0 and 4.0 mg/dL, indicating recurrent inflammation despite her being asymptomatic. Fatigue persisted, but no symptoms of giant cell arteritis were present, and temporal artery ultrasound showed no halo sign. Increasing prednisolone to 30 mg/day did not improve her condition.

Three months after her initial visit, primary hyperparathyroidism was diagnosed. Suspecting CPPD/PM, colchicine 0.5 mg/day was introduced, leading to the resolution of fatigue and normalization of inflammatory markers. Prednisolone was subsequently tapered.

Twelve months after her initial visit, while hospitalized for infectious enteritis and on prednisolone 7 mg/day, she developed right wrist pain. Ultrasound and X-ray imaging showed no CC in the wrist joint but revealed calcifications in the extensor tendons, leading to a diagnosis of calcific tendinitis. Prednisolone was increased to 15 mg/day, and symptoms resolved within two weeks.

The patient has since remained symptom-free on prednisolone 5 mg/day and colchicine 0.5 mg/day, with no recurrence reported during follow-up.

Case 4

A 79-year-old male patient with a history of mild cervical spinal cord injury presented with progressive joint pain and declining physical function. Several months prior to admission, he developed bilateral shoulder and knee pain, lower back pain, and appetite loss. His activities of daily living deteriorated, and he became unable to manage daily tasks 10 days before admission, prompting emergency transport to our hospital.

**Figure 4:**
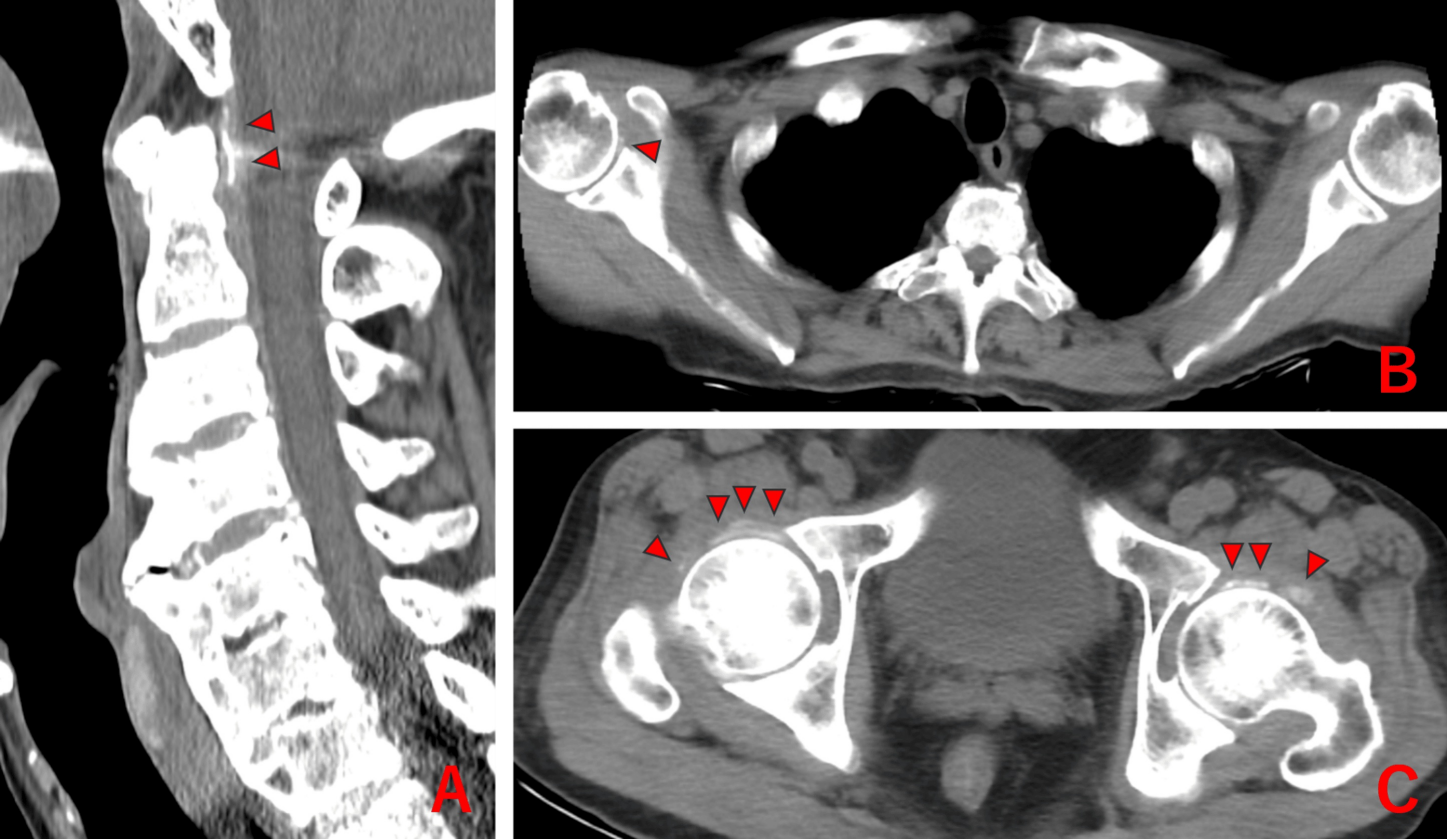
Axial vertebral region, right shoulder, and bilateral hip joints of Case 4 showing chondrocalcinosis (△)

At the initial evaluation, the patient exhibited severe motion-related pain in both shoulders and hips, mild motion-related pain in both knees, but no neck pain during rotation, peripheral arthritis, or lower extremity edema. Laboratory tests revealed elevated inflammatory markers (WBC of 11,100/µL, CRP of 16.5 mg/dL, and ESR of 124 mm/hr). Rheumatoid factor and anti-CCP antibodies were negative. Ultrasound showed severe bursitis in both shoulders, and plain CT revealed CC in the right shoulder, both hip joints, and around the atlantoaxial joint (Figure 4).

Polymyalgia rheumatica was suspected, and treatment with prednisolone 15 mg/day was initiated, necessitating hospitalization. The patient’s symptoms improved with therapy, and inflammatory markers decreased, allowing for discharge. However, CRP levels remained mildly elevated at approximately 0.5 mg/dL and did not normalize completely. When prednisolone was tapered to less than 15 mg/day, symptoms recurred. Given the presence of knee joint pain, seronegative RA was considered. Methotrexate at 4 mg/week was introduced, leading to CRP normalization. Prednisolone tapering was resumed, and at 12 months post-admission, rheumatoid factor and anti-CCP antibodies remained negative.

Despite initial improvement, the patient frequently missed doses, resulting in symptom relapses characterized by polymyalgia, unilateral wrist arthritis, and finger joint inflammation. The patient also reported frequent diarrhea after starting methotrexate and expressed concerns about steroid-related side effects, which contributed to poor medication adherence.

Consequently, prednisolone and methotrexate were discontinued, and treatment with iguratimod 25 mg/day was initiated. Following this change, the patient demonstrated improved adherence to therapy, with no further symptom relapses during follow-up.

## Discussion

We report four cases of polymyalgia presenting with CC in major joints, identified via CT imaging, and ultimately diagnosed as CPPD disease.

Case 1 involved a patient with a long history of RA who later developed polymyalgia, leading to a re-diagnosis of PMR. Despite steroid therapy, the patient experienced recurrent episodes of acute monoarthritis. Synovial fluid analysis revealed CPP crystals, resulting in a diagnosis of CPPD/PM. This case highlights the challenge of delayed diagnosis, as it took 16 years from symptom onset to the final diagnosis of CPPD/PM. The introduction of colchicine significantly reduced the frequency of arthritis episodes, emphasizing its therapeutic potential in managing such cases.

Pego-Reigosa et al. reported that CPPD/PM has distinct characteristics compared to PMR, including older age at onset, a higher prevalence of peripheral arthritis, more frequent osteoarthritis in the hands and knees, greater severity of knee involvement, and increased tendon calcification. Peripheral arthritis is significantly more common in the wrists, ankles, and metatarsophalangeal joints. However, while knee arthritis is also more prevalent in CPPD/PM, no significant difference has been confirmed. Thus, it is challenging to definitively attribute knee arthritis with polymyalgia to CPPD/PM [8]. Nonetheless, in cases like this one, where acute monoarthritis recurs, CPPD should be considered over PMR. Proactive joint aspiration to confirm the presence of CPP crystals is essential, and management should focus on colchicine and nonsteroidal anti-inflammatory drugs (NSAIDs) for effective control.

In Case 1, elevated hepatobiliary enzymes were observed during the polymyalgia phase. According to Pego-Reigosa et al., abnormal liver enzyme levels were reported in 19.4% of patients with CPPD/PM and 13.4% of those with PMR [8]. While reports of liver dysfunction associated with CPPD are limited, approximately 30% of patients with PMR or giant cell arteritis (GCA) experience liver dysfunction, which improves with treatment [10]. In this case, the elevated liver enzymes resolved following treatment, suggesting that the liver dysfunction was related to CPPD/PM.

In Case 1, lower extremity edema was observed during the recurrence of polymyalgia 37 months after discharge. Approximately 10% of PMR cases are reported to present with distal limb edema [11, 12]. In this case, the edema resolved rapidly following an increase in the steroid dosage, suggesting that it was associated with polymyalgia. This observation underscores the importance of recognizing that CPPD/PM can also present with distal edema.

Case 2 involved a patient diagnosed with CPPD/PM at the initial presentation based on the presence of CC in major joints on CT and responsiveness to NSAIDs, allowing for avoidance of prolonged steroid use.

In both Case 1 (22 months post discharge) and Case 2, neck pain during rotation was observed during the polymyalgia phase. As reported by Aouba et al. [6], this suggests that CDS may have contributed to polymyalgia. In Case 2, CC was also identified around the atlantoaxial joint on CT. However, CC around the atlantoaxial joint is observed in 34% of individuals over 60 years old and 49% of those over 80 years old, making it more prevalent than CC in other joints [13]. Therefore, such findings may be frequently observed in PMR, reducing their diagnostic significance compared to CC in the shoulders or hips. When considering polymyalgia due to CDS, assessing the presence of neck pain during rotation is crucial for accurate diagnosis.

On the other hand, both Case 1 and Case 2 exhibited lower-body polymyalgia, including hip pain and difficulty walking, which could not be explained solely by CDS. In the five cases of CDS presenting with polymyalgia reported by Aouba et al. [6], lower-body polymyalgia was absent. Thus, the absence of painful stiffness of the pelvic girdle has been suggested as a distinguishing feature between CDS and PMR. However, in our cases, lower-body polymyalgia and hip CC coexisted with CDS, suggesting that the combination of CDS and hip arthritis contributed to generalized polymyalgia. This indicates that distinguishing between CPPD/PM, including CDS, and PMR based solely on the distribution of polymyalgia may be challenging. Therefore, proactive imaging to confirm the presence of CC at sites of polymyalgia is essential for accurate differentiation.

Case 3 involved a patient with inadequate response to high-dose steroids typically used for PMR but who achieved symptom control with colchicine, consistent with treatment for CPPD/PM. Since the treatment strategies and prognoses for CPPD/PM and PMR differ, as demonstrated in Case 3, misdiagnosing CPPD/PM as PMR can result in excessive steroid use. Thus, distinguishing between the two conditions at an early stage is crucial [7].

In this case, the diagnosis of primary hyperparathyroidism prompted further investigation, leading to a diagnosis of CPPD/PM. Risk factors for CPPD disease include hyperparathyroidism, hemochromatosis, hypomagnesemia, and hypophosphatasia. The 2023 CPPD classification criteria highlight these as associated metabolic conditions [14]. Among these, hyperparathyroidism is particularly common, affecting approximately 2% of older adults [15]. Therefore, in patients presenting with polymyalgia and primary hyperparathyroidism, suspicion for CPPD/PM should be raised.

Case 4 initially presented with polymyalgia and knee symptoms, raising suspicion for seronegative elderly-onset RA. However, CT imaging revealed CC in major joints, leading to a final diagnosis of CPPD/PM.

Seronegative RA is another condition that is often mistaken for PMR [2], and CPPD/PM may also be misdiagnosed as seronegative RA. Krekeler et al. investigated the prevalence of CC in RA patients and found a higher detection rate of CC in seronegative RA compared to seropositive RA (32.3% vs. 16.6%) [16]. This suggests that a significant proportion of patients initially diagnosed with seronegative RA may actually have CPPD disease.

In distinguishing RA from PMR, the presence or absence of peripheral synovitis is useful [17]. However, CPPD/PM has also been shown to exhibit more peripheral arthritis compared to PMR [8]. Therefore, peripheral arthritis alone is insufficient to fully differentiate seronegative RA from CPPD/PM, and confirming the presence of CC via imaging is crucial. In cases like Case 4, where peripheral arthritis becomes more prominent over time, it is important to consider not only RA but also CPPD/PM as a possible diagnosis.

The relationship between CC in major joints and polymyalgia, as observed in Cases 1 to 4, has not been previously reported, making this the first documented case. One of the significant contributions of this report is the recognition of CC in the major proximal joints of the limbs, which aligns with polymyalgia and suggests that CPPD in these joints may be a key feature of the CPPD/PM pathology. While, as seen in Case 2 and in the report by Aouba et al. [6], upper-body polymyalgia may be caused by CDS, in cases like Case 3, where no neck rotation pain was present but symptoms were found in the shoulders and hips, support the idea that CPPD in these joints is the underlying cause of polymyalgia. While the significance of CC in the major proximal joints of the limbs has not been fully clarified, it appears to be an important factor, as it may contribute to the development of polymyalgia.

One of the key contributions of this report is the suggestion that the detection of CC within major joints may serve as a useful tool for differentiating CPPD/PM from PMR. Both CPPD/PM and PMR share polymyalgia as a primary symptom, but there is no gold standard test for diagnosis. It has been reported that the presence and location of peripheral arthritis can aid in distinguishing between the two conditions [8], and based on our clinical experience, peripheral arthritis often facilitates an easier diagnosis of CPPD/PM. This is likely because when peripheral arthritis is present, the clinical picture tends to resemble polyarthritis rather than polymyalgia, making it easier to consider CPPD diseases. Additionally, it is relatively straightforward to confirm CPP crystals and CC at the peripheral arthritis sites. However, when there is no peripheral arthritis and only polymyalgia is present, it becomes difficult to clinically differentiate CPPD/PM from PMR. In such cases, even when utilizing the 2023 CPPD classification criteria [14], a diagnosis may not be established if CPP crystals are not confirmed, which presents a barrier to further differentiation. Therefore, the detection of CC within major joints via CT is considered particularly useful for diagnosing CPPD/PM in cases with isolated polymyalgia and no peripheral arthritis.

Further research is needed to determine the diagnostic accuracy of CC within major joints in distinguishing CPPD/PM from PMR. According to Ottaviani et al., detecting CC in the acromioclavicular joint via ultrasound is a useful finding for differentiating CPPD/PM from PMR, with a sensitivity of 85.2% and specificity of 97.1% [7]. However, in the four cases we encountered, CC was not detected in the acromioclavicular joint on CT, and the CC identified in the shoulder was located in the glenohumeral joint. This difference is likely due to the distinct characteristics of ultrasound and CT. Ultrasound has the advantage of providing detailed images of superficial structures and is particularly effective for evaluating bursae and small joints, but it is challenging to visualize the internal structures of deeper major joints like the hip and glenohumeral joints.

## Conclusions

We encountered four cases of patients diagnosed with CPPD/PM who showed CC in the shoulder and hip joints on plain CT scans. The cause of polymyalgia in these patients is believed to be CPPD affecting the shoulder and hip joints. Given that the frequency of CC in the shoulder and hip joints is low in healthy individuals, these findings may be useful in distinguishing CPPD/PM from PMR. Further research is needed to explore this hypothesis.
